# Recent developments of optically stimulated luminescence materials and techniques for radiation dosimetry and clinical applications

**DOI:** 10.4103/0971-6203.42748

**Published:** 2008

**Authors:** A. S. Pradhan, J. I. Lee, J. L. Kim

**Affiliations:** Department of Health Physics, Korea Atomic Energy Research Institute, Yuseong, Daejeon, South Korea

**Keywords:** Accident/space/clinical dosimetry, optically stimulated luminescence, dosimeters, real-time on-line measurements

## Abstract

During the last 10 years, optically stimulated luminescence (OSL) has emerged as a formidable competitor not only to thermoluminescence dosimetry (TLD) but also to several other dosimetry systems. Though a large number of materials have been synthesized and studied for OSL, Al_2_O_3_:C continues to dominate the dosimetric applications. Re-investigations of OSL in BeOindicate that this material might provide an alternative to Al_2_O_3_:C. Study of OSL of electronic components of mobile phones and ID cards appears to have opened up a feasibility of dosimetry and dose reconstruction using the electronic components of gadgets of everyday use in the events of unforeseen situations of radiological accidents, including the event of a dirty bomb by terrorist groups. Among the newly reported materials, a very recent development of NaMgF_3_:Eu^2+^ appears fascinating because of its high OSL sensitivity and tolerable tissue equivalence. In clinical dosimetry, an OSL as a passive dosimeter could do all that TLD can do, much faster with a better or at least the same efficiency; and in addition, it provides a possibility of repeated readout unlike TLD, in which all the dose information is lost in a single readout. Of late, OSL has also emerged as a practical real-time dosimeter for in vivo measurements in radiation therapy (for both external beams and brachytherapy) and in various diagnostic radiological examinations including mammography and CT dosimetry. For in vivo measurements, a probe of Al_2_O_3_:C of size of a fraction of a millimeter provides the information on both the dose rate and the total dose from the readout of radioluminescence and OSL signals respectively, from the same probe. The availability of OSL dosimeters in various sizes and shapes and their performance characteristics as compared to established dosimeters such as plastic scintillation dosimeters, diode detectors, MOSFET detectors, radiochromic films, etc., shows that OSL may soon become the first choice for point dose measurements in clinical applications. A brief review of the recent developments is presented.

## Introduction

Optically stimulated luminescence (OSL) is a process in which a pre-irradiated (exposed to ionizing radiation) material when subjected to an appropriate optical stimulation, emits a light signal proportional to the absorbed dose. The wavelength of the emitted light is the characteristic of the OSL material. OSL is thus analogous to thermoluminescence (TL) process except that the stimulation is carried out optically rather than thermally and therefore competes with TLD, which has been popular for passive radiation dosimetry in clinical applications and in radiation protection. Like TL, the efficiency of OSL is a property of the solid state material but the OSL emission is highly influenced by the energy and the intensity of the stimulating optical beam. OSL, however, differs from other optical processes such as 1. radioluminescence or scintillation (in which the emission of light signal continues as long as ionizing radiation remains incident on the sample and the intensity is proportional to the dose rate); 2. photo-luminescence or fluorescence (in which the emission of light signal continues as long as a light beam is incident on a sample and the signal may or may not be influenced by the radiation); and 3. radio-photo-luminescence (in which on subsequent illuminations by an appropriate light, the emission of a light signal {of wavelength larger than the illumination light; Stoke shift} proportional to the amount of pre-irradiation takes place during an illumination due to the excitation and de-excitation of stable color centers and the same signal can be recorded any number of times from an irradiated sample whereas the resetting is done by a pre-established heat treatment). In OSL, there is no correlation between the wavelengths of stimulating light and the emitted light; the emitted light could be of longer or shorter wavelengths as compared to those of the stimulating light.

The credit for the discovery of OSL in radiation dosimetry should go to Albrecht and Mandeville,[1] who demonstrated that a photo-stimulation by a light of wavelength 410 nm results in an ultraviolet emission in x-ray–exposed BeO samples. This was attributed to the presence of doubly occupied electron traps, analogous to F-centers in alkali halides. Later, Braunlich *et al.* and Sanborn and Beard, as early as 1965,[2,3] used an infrared stimulation for the dose-dependent readout of TLDs, and the first few materials tested were Ce-, Sm-, and Eu-doped MgS, CaS, and SrS. Although these materials had been known to be converters of infrared into visible light, the mode of simultaneous stimulation and detection of an emitted light signal did not attract much attention till 1985, when Huntley *et al.*[4] demonstrated the use of green light from an Argon laser (514.5 nm) for the stimulation of quartz and feldspar for optical dating of sediments. This mode remained limited mainly to the field of dating until the observation of time by Markey *et al.* resolved optically stimulated luminescence in a highly sensitive α-Al_2_O_3_:C TLD material.[5] However, in some other TLD materials, the phosphorescence caused by an optical stimulation was shown to be useful for radiation dosimetry (e.g., BeO,[6,7] CaF_2_:Mn,[8] and CaSO_4_:Dy[9–11]). This use of optically enhanced phosphorescence had only a limited success due to its low sensitivity. Had the early observation by Tochilin *et al.*[7] (that the sensitivity of OSL would be considerably increased by directly measuring the fluorescence during stimulation rather than measuring the optically stimulated phosphorescence) been taken seriously, the usefulness of OSL for radiation dosimetry might have emerged much earlier. The main advantages of OSL over TL are 1. the sample can be read repeatedly as the optical stimulation depopulates the traps only partially (for shorter stimulations); 2. there exist a wide range of possibilities for stimulations and measurements by using different types and modes of light beams for an optical stimulation and measuring systems; 3. very narrow stimulating beams (a few tens of nm) could offer a high spatial resolution of a dose distribution in an OSL material; and 4. no heating to higher temperatures is needed for the readout of OSL and thus thermal changes influencing the sensitivity and thermal quenching of a luminescence signal (usually dominant in some TLDs due to heating) are avoided.

The acceleration in the interest and the developments in optically stimulated radiation dosimetry, as evident from the publications, appear to have occurred following a publication entitled ‘A radiation dosimetry method using pulsed optically stimulated luminescence’ by Akselrod and McKeever.[12] During the last 10 years, OSL has been one of the most widely studied techniques in the field of radiation dosimetry. As evident from the international debates,[13] it has already become a formidable competitor to the established technique of thermoluminescence dosimetry (TLD). In USA, Al_2_O_3_:C–based OSL dosimetry systems meeting the ANSI (American National Standards Institute) criteria are being used by some leading and nationally accredited service providers for personal dosimetry, replacing TLD.[14] This has also been introduced for personal dosimetry in some other countries.[15] Estimations have indicated that more than 25% of about 5 million badges in use in the world are in fact OSL dosimeters.[14] Apart from personal and environmental dosimetry, Al_2_O_3_:C OSL dosimeters have also been accepted by the National Council on Radiation Protection and Measurements (NCRP) for the dosimetry of astronauts and the habitable volumes of spacecrafts .[16] During the last few years, OSL has emerged as a real-time *in vivo* dosimetry technique for radiotherapy beams, apart from applications in mammography and CT dosimetry. This is because Al_2_O_3_:C–based OSL systems appear to possess the properties near to an ideal dosimeter, such as high sensitivity, high special resolution, availability in different shapes and sizes, no or few dependencies on beam parameters, capability of measurements of absorbed dose in real time for both photon and electron beams, and temperature independence for the ease of calibration and use. The availability of Al_2_O_3_:C has so far remained limited to a single source/supplier; but of late, attempts have been made and the monopoly of Al_2_O_3_:C for OSL appears to have been challenged by some other laboratories. Several new dosimetric materials and techniques have also been reported. For the past two decades, OSL has also been widely used for phosphor storage plates, and many new developments and applications are appearing in recent publications for two-dimensional imaging. There have been reviews on OSL,[14,17] but the present review deals with some more recent developments in radiation dosimetry.

## Concepts and OSL Terminology

**Figure 1:**
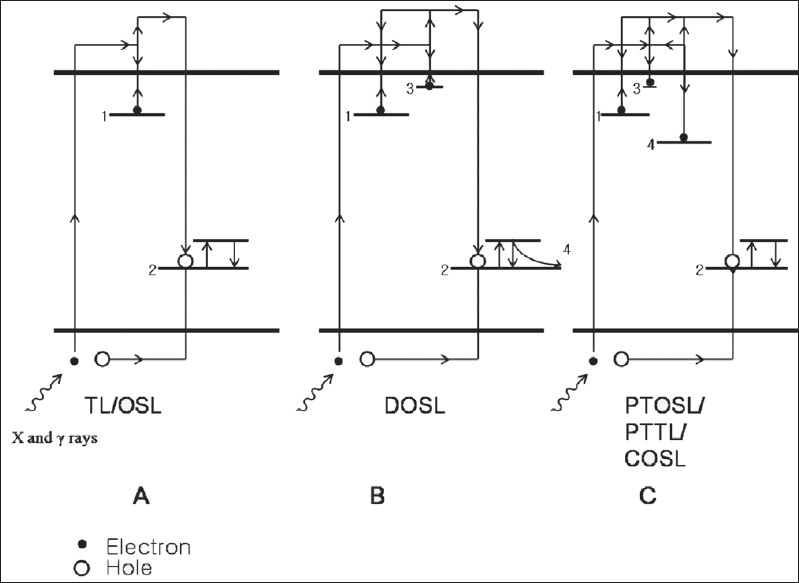
Simple representation of electron and hole ion pair production by ionizing radiation, movement (using arrows similar to those followed in books[14]), trapping, re-trapping, transfer between the traps, recombination, and luminescence emission. (A) Shows a simple case of TL or OSL with no intertransfer of the charge carrier among the traps. (B) Shows the delayed emission (DOSL) through photo-transfer via shallow traps (used in optically enhanced phosphorescence) and also if the luminescence center has higher lifetime (used in POSL, which is the other type of DOSL). (C) Shows the modes of photo-transferred optically stimulated luminescence (PTOS), photo-transferred thermoluminescence (PTTL), and cooled optically stimulated luminescence (COSL). 1- electron trap, 2- hole trap or recombination center associated with a luminescent center, 3- shallow trap, and 4- deep trap responsible for photo-transfer. For details, see text

Luminescence concepts in insulators and crystalline solids are best understood by an energy band model of the valence band (VB) and the conduction band (CB) separated by a forbidden band (FB). Without any thermal or optical excitation, the uppermost band of occupied electron states (VB) is completely full, and the states are occupied by the electrons originating from bound electrons of atoms. The next band (CB) of energy states is completely empty and is separated by a band gap (FB). In a material having perfect crystal symmetries, there is no electronic state between the top of the VB (the highest state of occupied bands) and the bottom of the CB (the lowest state of unoccupied bands). The reason why the unoccupied band is called the CB is the fact that an electron in the CB is freely mobile if it is excited by thermal or optical energy. The empty state in the VB (created by the excitation of electron to be in the CB) behaves as if it were a mobile particle with a positive charge called a ‘hole’ (a hypothetical particle). The crystal imperfections including point defects (intrinsic and extrinsic) created during the syntheses or doping are known to produce metastable localized states in the forbidden band. Some of these act as traps for the electrons and holes produced by the interaction of ionizing radiations (charged particle and X and gamma rays) with the material. A simplistic representation of the processes is shown in Figure 1, where the mobility of electrons in the CB is depicted by continuous arrows at different levels only for clarity and these should not be taken as discrete energy levels in the conduction band. Some of the electrons and holes produced by ionization could result in a radiative recombination in one of the two ways: 1. instantaneous recombination at the recombination center at stage 2 of [Figure 1A] after the production and during an irradiation to cause a radioluminescence (RL) or scintillation without getting trapped; and 2. recombination via metastable states where some of the charge carriers are trapped (electrons at stage 1 and holes at stage 2 of Figure 1A) and can remain trapped for long periods of time until sufficiently stimulated. Since RL is produced continuously during an irradiation, information about a radiation dose rate could be obtained in real time. On the other hand, a trapped electron (stage 1 of Figure 1A) can be released either by means of a thermal stimulation (by heating in TL) or by an optical stimulation (by shining light of appropriate wavelength in OSL) at any point in time after an exposure to ionizing radiation and can travel freely in the CB until it recombines with a hole. In TL, the stimulation of a pre-irradiated sample by heating to a fixed temperature results in the emission of a light signal proportional to the amount of ionizing radiation which peaks at fixed temperatures (as glow peaks), and the sample is reset for subsequent use. The temperature of the glow peak is related to the trap depth (higher the peak temperature, deeper the trap), from where the charge carriers trapped during the irradiation are liberated to result in a radiative recombination at stage 2 of [Figure 1A]. The amount of energy needed to release a charge carrier from a trap depends on the trap depth; deeper traps need more energy for stimulation. The released electron eventually recombines with a hole at the recombination center. The recombination center could itself act as a luminescent center, or the recombination energy could be transferred to excite a spatially related luminescent center which emits its characteristic emission while returning to its ground state.

Unlike a thermal stimulation for TL where all the charge carriers related to a glow peak are liberated on attaining a peak temperature and the information is lost in a single readout, an optical stimulation by low-intensity source is not able to sufficiently stimulate all the trapped charge carriers of the pre-irradiated sample in a single/short illumination and thereby provides a possibility of multiple readouts of an irradiated sample. The resetting for a subsequent use can be carried out either by using an established heat treatment or by an intense or long-duration optical bleaching by using a light source of appropriate wavelengths. The conventional OSL signal can be recorded by integrating the light emission for a fixed interval of time following stimulation. There have been several modes of stimulation and detection of light signals leading to varying terminology. Some of the common terms are CW-OSL (for continuous-wave optically stimulated luminescence), DOSL (for delayed optically stimulated luminescence), LM-OSL (for linearly modulated optically stimulated luminescence), POSL (for pulsed optically stimulated luminescence), PTOSL (for photo-transferred optically stimulated luminescence), and COSL (for cooled optically stimulated luminescence).[14]

**Figure 2:**
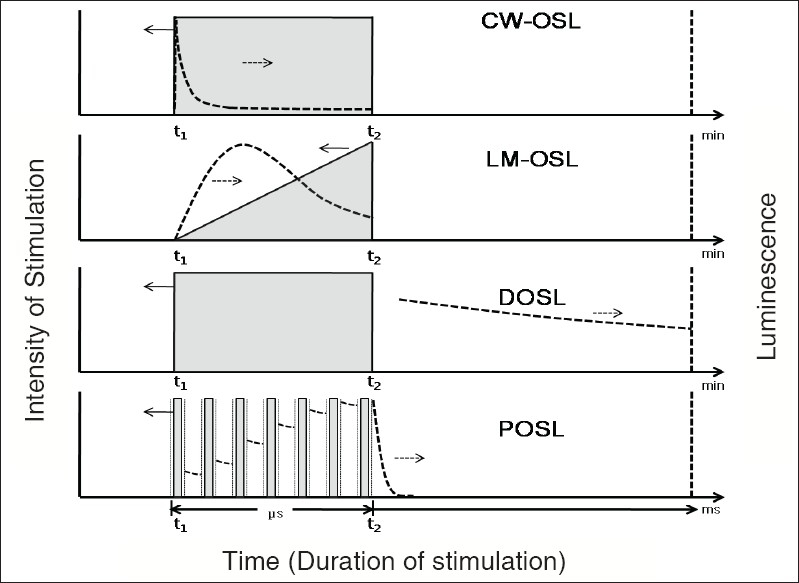
Representation of different modes of optically stimulated luminescence and its decay with time. Stimulation starts at time t_1_ and ends at t_2_ (for details, see text). For DOSL, the time scale in minutes is for optically enhanced phosphorescence through photo-transfer

‘Continuous wave optically stimulated luminescence’ is the simplest and the most straightforward OSL process (Figure 2, CW-OSL), in which a pre-irradiated material is stimulated by a light source of constant intensity, and the emitted luminescence signal of wavelengths different than the stimulation is recorded during a stimulation.[5] Unless specified otherwise, the term OSL is generally referred to CW-OSL. One of the most important requirements for OSL is that the wavelengths of a stimulating beam and emitted light have to be as apart as possible and the detector (usually a PM tube) should be optimized to record only the emitted luminescence with a minimum possible contribution (preferably nil) from the stimulating source. This is done by using appropriate optical filters both for the stimulating beam and the detector. Selection of optical filters is the most crucial parameter; and even with the best efforts, the complete avoidance of interference may not be possible. The shape of OSL decay (Figure 2, CW-OSL) is a function of material characteristic but can be altered by the intensity of stimulation and other experimental conditions. In general, when the crystal is stimulated by light to produce OSL, the emitted signal starts with an initial high value which decreases exponentially with time in a single exponential function or multiple exponential functions. CW-OSL continues to be the most sensitive mode of OSL, because the intensity of the emitted signal is the highest during a stimulation and there is no restriction on the duration of a stimulation or recording for an optimization of the signal-to-noise ratio. The OSL readout could be very fast (∼ s) because most of the OSL emission occurs immediately, at the start of the optical stimulation (switching the light source on).

‘Linearly modulated optically stimulated luminescence’ is similar to CW-OSL except that the intensity of the stimulating light beam is ramped and linearly increased (Figure 2, LM-OSL) instead of a constant intensity source of stimulation. The emitted signal is monitored during the ramped stimulation.[18] The light emission in the LM-OSL results in the form of peaks(like TL glow peaks) as a function of the readout time. This is because the distribution of the traps results in different photo-ionization cross sections for different wavelengths of stimulations. The release of the electrons from the traps depends on their photo-ionization cross section; the larger the photo-ionization cross section, the quicker is the release. By increasing the power of the stimulating light source, the release of electrons first increases and then reaches a maximum and thereafter decreases as they are no more available. The LM-OSL peaks have no correlation with the TL glow peaks. The traps related to a TL glow peak may or may not exhibit an LM-OSL in a sample exhibiting both the TL and OSL properties. A lower-temperature TL glow peak related to shallow traps may even exhibit much delayed peak on the LM-OSL plot if the related traps have lower photo-ionization cross sections. LM-OSL provides a way out to read different trap components with different photo-ionization cross sections.

‘Delayed optically stimulated luminescence’ should be a process in which the optically stimulated luminescence signal from a pre-irradiated material is measured after a stimulation light is switched off, i.e., the light detector (usually a photomultiplier PM tube) does not record any signal during a stimulation (Figure 2, DOSL). DOSL could be of two types: one in which the phosphorescence which is known to operate through the shallow traps (stage 3 of [Figure 1B])[6–11]; and the other in which the luminescence is delayed due to the extended lifetime (order of several tens of milliseconds) of the luminescent centers (stage 4 of [Figure 1B]).[5,12] The main source of the charge carriers for both optical stimulation types of a pre-irradiated sample is a large number of stable traps (stage 1 of [Figure 1B]), which reach the luminescence centers either through unstable (unstable at room temperature) shallower traps (stage 3 of [Figure 1B] for type 1) or without any photo transfer (stage 4 of [Figure 1B] for type 2).[10] In the former case, the charge carriers liberated from the stable traps (stable at normal temperatures) by the optical stimulation are re-trapped at the shallower/unstable traps, and a slow release of these charge carriers results in enhancing the phosphorescence to cause the delayed luminescence signal (Figure 2, DOSL) for duration up to several tens of minutes after an optical stimulation. The phosphorescence caused by an exposure to ionizing radiation through unstable traps in the form of an afterglow, subsides shortly after the exposure and has small intensity when compared to the photo-stimulated phosphorescence. In the latter case of the extended lifetime of the luminescent center, the charge carriers released by a stimulation reach the luminescence center and the emission could be delayed for durations of the order of a few tens of milliseconds (step 5 of [Figure 1B]). Recording of dose-dependent DOSL has the advantage of a complete discrimination between the stimulated light and the emitted signal[12] with no limitation on the energy and the intensity of a stimulation.

‘Pulsed optically stimulated luminescence’ is a form of DOSL in which the luminescence is recorded intermittently following a stimulation by very short pulses (of duration of the order of hundreds of nanoseconds) with a frequency of about thousands of hertz (time gap of the order of a few hundreds of microseconds between the two pulses). The optical stimulation is thus turned on and off periodically, and the OSL in the form of a delayed emission or the afterglow between the pulses (when optical stimulation is turned off) is recorded and added up to the previous value until an equilibrium value for a POSL signal is attained (Figure 2, POSL). The detector (PM tube) is blocked for a period of the order of a few tens of microseconds — much longer than the duration of the stimulating pulse. No signal is detected during stimulation. The detection of the emitted OSL signal and the stimulation are thus separated by time discrimination, and the dependence on optical filtration is minimized. The POSL signal could be increased by increasing the power of the stimulating laser source and decreasing the pulse width. Short and intense pulses are able to result in larger POSL signals. The OSL signal is due to the luminescence from the centers with a lifetime of about 35 ms. Unfortunately, the background signal (nonradiation-induced signal) is not completely independent of the stimulation and increases considerably by increasing the power of the stimulating laser pulses.[12] Also, the presence of luminescence centers with longer lifetimes (of the order of a few tens of milliseconds) is not common in other materials. However, POSL technique has been successfully demonstrated for the use of α-Al_2_O_3_:C in radiation dosimetry by using pulses of frequency of 4 kHz and a pulse width of 300 ns for a stimulation and by blocking the PM tube for 15 *μ*s[12] during which no signal is recorded.

The term ‘photo-transferred optically stimulated luminescence’[19,20] is analogous to the popular term ‘photo-transferred thermoluminescence’ (PTTL) which is prevalent in TLD. In a pre-irradiated sample, the traps related to the main OSL and/or dosimetric TL glow peaks are first emptied (by a readout treatment) without affecting the deep traps. On an illumination by light of proper energy, the charges from the deeper traps (stage 4 of Figure 1C) are evicted and result in a light signal either by a direct radiative recombination at the luminescence centers or when routed via shallower (unstable) traps. This luminescence signal, which is considered to be due to the transfer of charges carriers from deep traps, has been termed as PTOSL. Obviously, the PTTL signal (TL from the repopulated dosimetric glow peaks) has to be much less than the PTOSL because the PTTL is only from a small fraction of the charge carriers liberated from the deep taps, whereas a much larger fraction of the charge carriers liberated from the deep traps has the possibility to cause PTOSL.[21] The PTOSL provides a means/technique to use the deep traps (usually very stable) for radiation dosimetry.

‘Cooled optically stimulated luminescence’ was introduced by Miller *et al.*[22] and is a misnomer of PTTL with the only difference that heating for recording a COSL is carried out by bringing the cooled samples from very low temperatures (e.g., liquid N_2_ temperature) to room temperature. For COSL readout, the sample after an irradiation to a known dose is cooled down without any optical or thermal stimulation. The optical stimulation following an irradiation at room temperature is carried out at lower temperatures (e.g., liquid N_2_ temperature). While at a low temperature, the empty shallow traps are populated by the transfer of charge carriers from the traps filled due to an irradiation (stage 1 of Figure 1C), including the deep traps (stage 4 of Figure 1C) due to photo-stimulation. The shallow traps (stage 3 of Figure 1C) populated by an optical stimulation at a low temperature release the charge carriers while attaining room temperature, and a recombination of these charge carriers at a recombination center results in a dose-dependent light signal called COSL. COSL does provide a technique for a TL readout without heating the TLDs. The PTTL and COSL properties cannot be generalized for all materials as these are material specific.[23]

## Characteristics of OSL Materials for Radiation Dosimetry

### Al_2_O_3_

α-Al_2_O_3_:C (α-Al_2_O_3_:C and Al_2_O_3_:C should be considered to be the same until mentioned otherwise) was developed as an ultra-sensitive TLD material. It suffered a setback due to its extreme sensitivity to daylight for erasing the radiation-induced information, a high thermal quenching resulting in a steep reduction in its sensitivity to increasing heating rates during TL measurements, a reduction of its efficiency with increasing linear energy transfer (LET) of radiation, and a UV-induced TL sensitivity.[24] The intrusions drawn from its light sensitivity and photo-transformation of color centers provided a tool for new research leading to OSL. The erasing of the dosimetric TL glow peak of the irradiated Al_2_O_3_:C on exposure to light led to a clue for establishing a correlation between the TL peak and a stable OSL signal. Its chemical stability (high melting point) and wide forbidden gap (9.5 eV) allowing a large variety of stable traps and color centers opened up the possibility of an engineering synthesis of a variety of OSL dosimeters. It was found that the main luminescence produced by the F centers has a lifetime of about 35 ms,[5] and this led to the POSL technique,[12] which later culminated in the development of a popular Launder's Luxel Personal Dosimetry system. An equally sensitive and popular system based on the CW-OSL technique was also developed by Launder and named as InLight Personal Dosimetry system. Al_2_O_3_:C attracted worldwide attention, and its availability appears to be no more restricted to a single source as several others have also demonstrated the synthesis of this material.[25,26] Pure (un-doped) Al_2_O_3_ and that doped with other dopants such as Si, Ti, etc.,[27,28] did not exhibit enough OSL sensitivity. From these studies, some inference can be drawn on the role of C in Al_2_O_3_:C.

Al_2_O_3_:C is very sensitive to light, and therefore great care has to be taken by making a light-tight packing of the dosimeter and by following appropriate procedures for a readout and handling in dim light. Recent studies by West *et al.*[29] have shown that there are two opposite effects of exposure to sunlight, viz., 1. erasing of the radiation-induced dosimetric information of an irradiated sample and 2. induction of signal. An equilibrium was found to occur at about 15 mGy, implying that all dosimeters whether irradiated or un-irradiated would exhibit a signal of about 15 mGy equivalent to ^60^Co gamma rays. This finding has great relevance in radiation dosimetry and needs a re-look for the parameters of quality assurance.

The other problem with the use of Al_2_O_3_ is the large variation of the properties from sample to sample obtained from a single crystal and from batch to batch. However, it is easy to correct these variations, and the use of Al_2_O_3_:C continues to grow for dosimetry, even in mixed fields of low and high LET radiations in spite of the numerous intricacies. Like TL, an OSL response is also known to decrease with increasing LET of radiation and is also dependent on the charge, mass, and energy of a high-energy charged particle (HCP). A decrease in the OSL efficiency is related to the nonuniform distribution of the physical events created by the charged particles′ path, producing high doses along the vicinity of a track.[30,31] The OSL efficiency of different HCPs was observed to depend not only upon the linear energy transfer (LET) of the HCP but also upon the sample type (due to batch to batch variation), e.g., single crystal chip or Luxel™ type (with powder between two plastic layers); and the luminescence method used to define the signal, e.g., CW-OSL initial intensity (OSL integrated for the first few seconds after t_1_, [Figure 1] CW-OSL), CW-OSL total area (OSL integrated for 300 seconds after t1, [Figure 1] CW-OSL), or POSL. Besides the change in the OSL response, the nature of the OSL decay curves has also been found to change with the LET of a radiation, as well as for high doses of a low-LET radiation. Table 1 shows the OSL efficiency of Al_2_O_3_:C Luxel™ type dosimeter tohigh-energy and heavy charged particles relative to gamma rays for the readouts in three modes: 1. the initial CW-OSL, 2. integrated CW-OSL readout, and 3. POSL readout.[16,31] However, the response to high-energy protons was found to be similar to that for beta or gamma rays.[16] One of the exciting new findings has been the observation of a 355-nm (UV) emission (lifetime <t7 ns, dominant in the initial OSL readout), in addition to the commonly known 420-nm emission (lifetime 35 ms, responsible for the POSL signal and dominant in the integrated CW-OSL signal), and the change of the ratio of UV and 420-nm emission with LET. These findings are going to be of great importance for the dosimetry of mixed field of low- and high-LET radiation, especially for dosimetry in space, which is characterized by soup of charged particles over wide energy ranges and with varying fluxes. It may be noted that the radiation fields encountered by the astronauts change significantly with the location of the spacecraft; and to ensure the safety of the astronauts from the effects of radiation, the use of integrating personal dosimeters or area monitors is essential.

**Table 1:** OSL efficiency of Al_2_O_3_:C Luxel™ type and single-crystal dosimeter to high energy heavy charged particles relative to gamma rays for the readouts in three modes: 1. the initial CW-OSL (integrated for the first 3 seconds), 2. integrated CW-OSL readout (integrated for 300 seconds), and 3. POSL readout[16,31]

*Beamion*	*LET (keV/μm in water)*	*Initial CW-OSL Luxel™ SC*	*Integrated CW-OSL Luxel™ SC*	*POSL Luxel™*
H	0.22	0.98 –	0.91 –	–
H	0.413	1.11 –	0.95 –	–
H	0.96	1.10 –	0.93 –	–
He	2.26	1.37 1.01	1.00 0.83	0.81
C	11.20	1.32 0.73	0.76 0.53	0.60
Si	55.70	0.85 0.42	0.47 0.33	0.38
Fe	193.00	0.64 0.38	0.38 0.30	0.31

### BeO

One of the most promising OSL materials to compete with Al_2_O_3_:C appears to be BeO. Recent studies indicate that a nearly tissue-equivalent and a low-cost commercially available BeO material hasmuch larger potential than that foreseen so far. It is a pity that since 1970,[6] little attention was paid to the OSL in this material. The main reasons appear to be the earlier results indicating a poor DOSL sensitivity (lower limit of detection above 10 mGy) and the apprehension of its toxicity. However, BeO did find a large-scale application as a personal TL dosimeter[32] in Italy, and this was possible by overcoming the apprehension to its toxicity. It is now well known that the solid/sintered form of BeO (free from dust) can be handled with practically no health hazards. This material has mainly been used in the electronic industry on a very large scale as a heat sink because of its unique property of high electrical insulation and a very high thermal conductivity to withstand very high temperatures, up to 2000°C. Following the success of Al_2_O_3_:C, BeO re-attracted attention for radiation dosimetry using OSL. The wide separation of emission (in UV range peaking at 335 nm, the same as its TL and RL emission) and a stimulation (in the visible range peaking at 435 nm) provided an opportunity for reducing the interference in OSL measurements. One of the most fascinating findings[19] has been the demonstration of no correlation between intense TL glow peaks and the OSL signal. In BeO, the main TL glow peak at 220°C (main dosimetry peak), which also is very sensitive to an exposure to visible light / sunlight (light-induced fading and the induction of a signal like that of Al_2_O_3_:C), had no impact on its OSL signal. The OSL signal recorded by a blue-green (420-550 nm) light stimulation remained unchanged in the presence or the absence (after heating up to 280°C to completely remove the 220°C peak) of a 220°C TL peak.[19] Although the 220°C TL peak disappeared after a blue-green light stimulation, the charge carriers released from the traps related to the 220°C glow peak did not contribute to OSL. The higher-temperature TL glow peak at 340°C, which had a much smaller light sensitivity than the 220°C TL peak, was found to be responsible for the OSL signal in BeO. This brings out an inference that the traps related to the TL glow peaks which are sensitive to light may not contribute to OSL.

Efforts have been made to improve the measurement of low doses by using an OSL of BeO. Doses as low as 5 mGy (linear range, from 5 mGy to tens of Gy) could be measured from BeO ceramic disks (Thermalox 995, Brush Wellman, Inc., US) of ∼1 mm thickness and 4 mm diameter[19] by using a commercial OSL (Riso) reader. The OSL sensitivity of BeO was found to be smaller than that of Al_2_O_3_:C for the use of LM-OSL[33] and frequency-modulated pulsed OSL[34] methods at a dose level of 100 mGy. By using intense blue light–emitting diodes for the stimulation for the CW-OSL, doses as low as 20 μGy were measured.[35] More recently, by improvements in the measurement technique by reducing the distance between the detector (PM tube) and the sample (stimulating from one side and detecting the emitted signal from the opposite side of the disk), doses as low as a few μGy could be measured by Sommer.[36] BeO obtained from different sources is known to exhibit variation in its sensitivity by about a factor of 3,[37] and also impure samples are found to exhibit a much higher sensitivity than do pure samples.[7] The OSL properties of BeO clearly emphasize a strong need for preparing BeO doped with appropriate activators. Although BeO ceramics have been synthesized,[38] efforts for making OSL-grade BeO are still to be noted.

### MgO, Mg_2_SiO_4_:Tb, LiAlO_2_:Tb, and other Tb-activated silicates

MgO has been another attractive material because of its very high thermal stability (high melting point) and near tissue equivalence, similar to Al_2_O_3_ and BeO. UV and light sensitivity of an Fe-doped MgO material has been known for some time,[39] but OSL on irradiation to ionizing radiation was recently demonstrated by Boss *et al.*[40] in Tb-doped MgO. Doses above 1 mGy (linear up to 10 Gy and beyond) could be measured by using green LEDs for stimulation. This indicates that MgO:Tb, being a near tissue-equivalent material, has the potential for OSL dosimetry, and continued efforts are needed in this direction.

OSL of Tb-activated silicates and aluminates has also been reported by Masson *et al.*[41] Near tissue-equivalent LiAlO_2_:Tb and Li_2_Al_4_O_4_:Tb OSL materials, with an added advantage in a mixed field dosimetry of neutrons and gamma rays, have been prepared. The presence of Li makes it neutron sensitive by using the high thermal neutron cross-section of the ^6^Li content. The use of a ^7^Li-enriched (depleted in ^6^Li) material would make it neutron insensitive. Unfortunately, the OSL responses of Mg_2_SiO_4_:Tb, LiAlO_2_:Tb, and other Tb-activated silicates were found to be much smaller than MgO:Tb. In spite of their near tissue equivalence, these Tb-activated silicates and aluminates may not find much utility in radiation dosimetry unless remarkable success is made in the process of their preparation.

### ABF fluorides

KMgF_3_:Ce ABF fluoride (A=K, B=Mg, and C=F)[42,43] was found to exhibit OSL sensitivity about 10 times higher than that of the commercial Al_2_O_3_:C, for its UV (360 nm) emission for a stimulation by blue LED (470 nm, d0 l25 nm). In spite of a very high OSL sensitivity, it appears not to have given rise to much fascination mainly because of its high self-dose (∼1.5 *μ*Gy/h) due to the natural abundance of the ^40^K isotope and non–tissue equivalence (Z_Eff._ = 14.7). NaMgF_3_:Ce[42] with its OSL sensitivity comparable to that of Al_2_O_3_:C for its UV (280 nm) emission for a stimulation by blue LED, was found to exhibit a severe fading on post-irradiation storage.

Among the ABF fluorides, a new development of NaMgF_3_:Eu^2+^ has been reported by Dotzer *et al*.,[44] which appears to have a high potential for personal and environmental radiation dosimetry. The replacement of activator Ce by Eu (0.2%) appears to have had a great impact on the characteristics of the NaMgF_3_ (Z_Eff._ = 10.4) near tissue-equivalent material. It exhibited a linear dose response covering a dose range of more than 8 decades(studied linear dose range from a few *μ*Gy to 100 Gy) with the lowest detection limit of 130 nGy. The measurements carried out on a Riso reader showed that its OSL sensitivity could be significantly enhanced by a proper combination of the filters, and its post-irradiation storage fading could be improved by optimizing sintering and quenching treatments. It is evident that NaMgF_3_:Eu^2^ with its Z_Eff_ lower than that of Al_2_O_3_:C and very high OSL sensitivity would be a real breakthrough if this material also were to withstand the tests of chemical stability and dosimetric ruggedness.

### Ammonium salts and alkali halides

Generally, materials rich in their hydrogen content disintegrate chemically upon heating and are hence not suitable for TL. Since no heating is required in OSL, a material with good OSL properties and low melting point could be used directly as an OSL dosimeter or could be mixed with low melting point materials. Dosimeter materials rich in H_2_ content are preferred for neutron dosimetry. Ammonium salts such as thallium-doped NH_4_ Br and (NH_4_)_2_SiF_6_ have been found to exhibit significant OSL with detectable signals for doses as small as 5 *μ*Gy,[45] but the OSL signal faded by 80% within 10 hours after exposure to radiation and caused sever limitations.

Among alkali halides, the OSL of KCl and KBr has made some noticeable impact.[46] KBr:Eu[47] was found to exhibit about 10 times higher OSL sensitivity and smaller fading than KCl:Eu. The most striking features have been the dependence of fading on 1. the dopant concentration (smaller fading rate for lower Eu concentrations) and 2. the energy of ionizing radiation (decrease in the fading rate with increasing energies of x-rays from 20 kV to 40 kV). These were proposed to be good candidates for two-dimensional imaging in medical diagnostics, similar to that of BaFX:Eu. Both KCl:Eu and KBr:Eu suffer from the drawback of self-irradiation due to the natural abundance of ^40^K to limit their applications.

### Y_3_Al_5_O_12_ (YAG)

Yttrium aluminum garnet (YAG) is a laser material and is widely used for a variety of optical properties. YAlO_3_:Mn was shown[48] to exhibit OSL by using a blue-green (450-500 nm) light stimulation. Several other high-Z_Eff_ materials like RbCdF_3_:Mn and RbMgF_3_:Mn[49] have also been reported to exhibit very intense OSL, but their utility for radiation dosimetry also appears limited. Recently, Kulkarni *et al*.[50] developed an OSL material (Y_3_Al_5_O_12_:C). The samples prepared in the presence of a graphite liner (under high vacuum) exhibited intense OSL. It was shown to have a linear dose response in the range 10 mGy to 100 Gy. Absence of any fading within 2 months of post-irradiation storage is the most attractive feature of Y_3_Al_5_O_12_:C.

Table 2 compares the main OSL characteristics of some OSL materials. It should be noted that most OSL materials (except Al_2_O_3_:C, BeO, and YAG) suffer from the drawback of post-irradiation storage fading even when stored in the dark.

**Table 2:** Main characteristics of some optically stimulated luminescence materials

*OSL material [Ref]*	*Rel. OSL Sensitivity*	*Linear dose range wavelength range/peak (nm)*	*Stimulation wavelength (nm)*	*Main emission wavelength (nm)*	*Fading rate*	*Z Eff; (Tissue =7.4)*
AI_2_0_3_:C[12]	1.00	μGy-10Gy	450-550	∼420	<5%/y	11.3
BeO[36]	∼1.00	μGy-10Gy	∼ 435	∼335	6% in 1st 10 h and then nil	7.2
MgO:Tb[40]	∼1.00	100μGy- 10Gy	500-560	375, 420, 440, 470, 500, 650	43% in 1st 36 h and then nil	10.8
NaMgF_3_:Eu[44]	∼10.0	μGy -100Gy	∼ 470	360	40% in 1st 24 h and then nil	10.4
KMgF_3_:Cei[42,43]	∼10.0	1μGy- 10Gy	∼ 470	∼360	High and ^40^K Self-irradiation.	14.7
Li_2_AI_2_0_4_:Tb[41]	∼0.01	200μGy- 10Gy	<532	370, 420 and 440	50% in 1st 50 h	9.74
Mg_2_SiO_4_:Tbi[41]	∼0.11	30μGy- 10Gy	<532	370, 420 and 440	30% in 1st 10 h and then 0	11.23
Mg_2_SiO_4_:Tb,Co[41]	∼0.08	40μGy- 10Gy	<532	370, 420 and 440	30% in 1st 10 h and then nil	11.23
KCI:Eu[46,47]	∼1.00	100μGy- 10Gy	500-560	350-480 and 560-700	High and ^40^K Self-irradiation	18.1
KBr:Eu[47]	∼1.00	100μGy- 10Gy	500-560	350-480 and 560-700	High and ^40^K	31.76
(NH_4_)_2_SiF_6_:TI[45]	∼0.02	> few mGy	470	300-370	Self-irradiation 80% in 1st 10 h	10.31
Y_3_AI_5_O_12_:C[49]	∼0.10	10mGy- 100Gy	500-560	350-480 and 560-700	Negligible in 2 months	33.81

## Radiation dosimetry using OSL of phosphor storage plates

BaFBr:Eu phosphor storage plates (PSPs), widely used in computed radiography, exhibit dose-dependent photo-stimulated luminescence signal which increases linearly with dose over a range of several decades. The plates are generally composed of a thin coating of BaFBr:Eu phosphor grains of size 150 *μ*m on a 200 to 300 *μ*m thick flexible plastic sheet. When exposed to ionizing radiation, the PSP stores the energy as a latent image until stimulated to emit blue light (390 nm peak wavelength) as a luminescence signal on scanning (stimulating) by a red (632 nm) laser. PSPs have not only been used in computed radiography but also in several other applications in radiation therapy — for portal imaging, dosimetry, and routine check of dosimetric parameters, e.g., field size, flatness, symmetry, etc., for quality assurance to replace the use of classical films (also in autoradiography of tracers in tissue samples and in x-ray diffraction crystallography). Yamadera *et al.*[51] explored the development of a personal dosimeter based on PSP. In spite of a very high effective atomic number and fading in response to post-irradiation storage, PSP could be successfully used to provide information on exposures during diagnostic x-ray examinations. The linearity in the dose response in the range 0.1 to 50 mGy was of special interest in personal dosimetry, although the response was noted to be linear up to 10 Gy. A very high sensitivity (about 103 times higher than that of the film dosimeters which are commonly used for personal dosimetry) attracted its use as a personal dosimeter.[52] The photon energy response of PSP for diagnostic x-rays of effective photon energies in the range of 12 to 120 keV was found to be as good as that of film dosimeters by using a combination of the responses under metal filters of Al, Zn, Cu, Cd, and Pb of varying thicknesses from 0.1 to 1 mm. PSPs were also found to be a useful tool for measuring doses from beta sources. The response to ^147^Pm beta rays was found to be higher than to ^90^Sr and ^85^Kr beta rays by 68% and 35% respectively.[53] The utility of these dosimeters has now been extended[54] to cover neutron fields by sandwiching a PSP between polythene plates for using recoil protons and between nylon plates for using the ^14^N(n,p)^14^C reaction for detection of neutrons. From this, it is evident that PSP phosphor plates, not uncommon in medical institutions, can also be used for radiation dosimetry.

## Retrospective accident dosimetry using OSL of electronic components of mobile phones and ID cards (fortuitous dosimeters)

Studies of luminescence techniques have shown that materials which are not produced for dosimetric purposes (nonsynthetic materials) may also exhibit OSL and/or TL comparable to synthetic materials. Bricks and other building materials have been used for dose reconstruction in cases of several radiation accidents, including Hiroshima and Nagasaki and Chernobyl. Of late, there has been growing concern for the estimation of radiation doses for nonradiation workers (members of public) in the event of a radiological accident, especially in view of the potential threat of a dirty bomb by terrorist groups aiming at civilian targets. Such a situation cannot be covered by planned radiation dosimeters such as personal dosimeters or area monitors installed at some strategic locations. The doses in these events may not be as high as those of a nuclear accident but may be of great nuisance through creating panic. In cases of unexpected situations, not only are the encountered doses low enough to escape the range of remotely installed area monitors, but there is also the need for an evaluation of the doses in the shortest possible time by using well-defined objects because the delay in communicating the information on the risks enhances the disruption of normal life because of the resultant apprehensions. This also needs tracking of the sources and the culprits by radiation dosimetry at the earliest possible time. It becomes important to be able to measure much smaller doses as quickly as possible, and this enhances the search for sensitive and most commonly used items. OSL of several electronic components has been reported to be sensitive and to offer much faster measurements than TL.

Electronic components of mobile phones and ID cards have been evaluated for this purpose as these are considered to be carried by most individuals all the time. Goeksu[55] appears to have taken a lead to demonstrate that OSL in chip cards (health-care ID card or a phone card) could be used as a dosimeter. Optical stimulation by 800 nm (infrared stimulated luminescence, IRSL) and an emitted signal in the range of 320 to 650 nm were recoded as a measure of a dose. Low dose estimation was limited by a zero dose response which was about 100 mGy. The OSL response of the chip cards was linear in the range 250 mGy to 5 Gy with a stable signal at ambient temperatures. Subsequently, memory chip modules from ID cards were evaluated by Mathur *et al.*,[56] who studied blue stimulated luminescence (BSL). Stimulation was done by using blue diodes in addition to an IRSL. The minimum detectable dose by using BSL was found to be 20 mGy, with a linear dose response up to 10 Gy. These studies clearly proved the superiority of OSL over TL, in which samples exhibited a large zero dose signal, which was absent for OSL. Very recently, Inrig *et al.*[57] demonstrated the potential of the electronic components of cellular phones and similar devices for use as a fortuitous dosimeter with a linear response from 5 mGy to 100 Gy (possibly beyond) by using 470-nm stimulation for emission in the range of 290 to 370 nm. The rectangular resistors of size 1×0.5 mm and above were found to exhibit high OSL sensitivity from their white porcelain substrates (typical composition of 97% Al_2_O_3_ and 3% SiO_2_) on tan underside which was uncoated. The radiation-induced OSL signals of the resistors were found to be similar to those of the standard Al_2_O_3_:C dosimeter. The OSL exhibited both short-term and long-term fading to an extent of about 50% within 10 days. A mock experiment of evaluating doses was carried out by affixing cellular phones and a commercial electronic dosimeter (ED) on an anthropomorphic phantom and by subjecting them to gamma ray exposures from ^137^Cs and ^60^Co discrete source for 3 to 4 days. The resistors from the cellular phones were removed after the exposure, and the doses were estimated from the OSL readouts by being corrected for the fading. The estimated doses were within 0.02 to 0.15 Gy, and the accuracy was within 20% against the EDs. Since the OSL measurements are supposed to be quick, this study[57] appears to have opened up a feasibility of dosimetry and dose reconstruction using the electronic components (as fortuitous dosimeters) of gadgets of everyday use in the event of such unforeseen situations.

## Clinical applications

OSL has emerged as a powerful tool for the measurement of ionizing radiation in clinical applications ranging from intricate radiological diagnostic exposures to a variety of modern radiation therapy treatments. As mentioned earlier, digital imaging systems using OSL of PSPs have already replaced the radiographic films in computed radiography; in addition, successful attempts have also been made to use them in radiation therapy as an inexpensive tool for digital portal imaging of megavoltage beams, verification of intensity-modulated beams,[58,59] proton beam dosimetry,[60] and routine check of dosimetric parameters, e.g., field size, flatness, symmetry, etc., for quality assurance; and in several other applications where radiographic films were used as a passive detector. The use of mobile PSP cassettes (analogous to film cassettes) is an example of the application of OSL of PSPs for digital imaging, especially in cases where electronic portal imaging systems cannot be used. Other recent studies and applications of OSL are centered around the use of OSL from tiny crystals of Al_2_O_3_:C as a point detector. A probe of Al_2_O_3_:C of the size of a fraction of a millimeter could measure doses in the range of mGy to several Gys with high precision. Detectors based on Al_2_O_3_:C have become available in different shapes and sizes.[61,62] Two modes of OSL measurements used in clinical applications are 1. passive or integrating mode, in which the detectors are exposed to radiation and the readout is carried out at a later time (like TLDs); and 2. real-time or on-line mode, in which the readout is carried out during exposure to radiation by transporting the light signal through an optical fiber to provide the dose rate (like plastic scintillation dosimeters, Si diodes, and MOSFET transistors). The information on total dose becomes available through OSL measurement immediately after an exposure. For *in vivo* medical dosimetry, OSL system based on Al_2_O_3_:C appears to offer high accuracy, a possibility of fast measurements, a high special resolution in view of the small size of the detector probe, no or few dependencies on the beam parameters, and ease of use and calibration. The main aspects of the passive and on-line modes are presented below.

### Passive or integrating mode of OSL of Al_2_O_3_:C for the measurements of central-axis and depth dose curves, surface doses and quality assurance parameters of radiotherapy beams

Passive or integrating mode of OSL of Al_2_O_3_:C has been widely evaluated for clinical dosimetric measurements of high-energy photon and electron beams.[62–65] The uncertainty of a single OSL measurement estimated from the variance of a large sample of dosimeters irradiated with the same dose was found to be 0.7% for 6-MV photon beams. The differences between the measured and the expected doses were ≤0.7% for a depth in the range of 1.5 to 10 cm in a water phantom.[61] Similar results were obtained for electron beams of energies 6, 9, 12, 16, and 20 MeV and a photon beam of energy 18 MV.[62] In a water phantom, central-axis depth dose curves obtained by using plastic disks of 0.3 mm thickness and a diameter of 7 mm (containing Al_2_O_3_:C powder grains of size <105 *µ*m) for 6-MV and 18-MV photons and 6-, 9-, 12-, 16-, and 20-MeV electrons were in agreement with the ionization chamber values to within 1%, with a precision better than 0.7%. Al_2_O_3_:C OSL dosimeters, with a linear response up to several Gy, are able to do the same as that TLD can do, in addition, they provide a means of verification and re-verification of the first measurement (as a permanent record of the dose information till resting) because OSL dosimeters can be read again and again. In the subsequent readouts, only a small amount of information (0.05% per reading) is lost, which can be easily corrected by using pre-established calibration factors. The OSL response of the light-protected dosimeters is very stable for several months (fading, less than 2%). Resetting for subsequent use[61] can be done by the simple treatment of min exposure to a tungsten-halogen lamp or by exposure to room light for several hours. A transient signal of half life 0.8 min was observed but a wait of 8-12 min after the end of an irradiation was found sufficient to cause no further change in the response. No significant energy dependence for 6-MV and 15-MV photon beams and 6 to 20 MeV electron beams has been observed. For ^192^Ir gamma rays, Jursinic[64] reported an over-response of 6%. Yukihara *et al.*[63] observed the relative response for 18-MV photons to differ by only about 0.5% from that of 6-MV photons. For doses up to 3 Gy, they did not observe any change in the sensitivity for a reuse after a resetting; but at high doses, a small change was observed, which could also be easily corrected by a method of repeated reference exposure.[63] This enabled them to keep the uncertainty within 1% for doses up to 10 Gy. Unlike diodes, there is no dose rate dependence. Differences in the results of the energy dependences of the photon and electron beams reported by different authors have been discussed by Yukihara *et al.*[63] Schembri and Heijmen[65] reported a difference of 3.7% between the responses to 6-MV and 15-MV photon beams, whereas Yukihara *et al.*[63] reported a difference of only 0.05%. For photon beams, the results of Yukihara *et al.* were in agreement with the results of Jursinic[61] and Aznar.[58] Table 3 shows the recent results for the photon energy dependence of Al_2_O_3_:C. For electron beams, Schembri and Heijmen[62] reported an under-response of 3.6% as compared to the response to photon beams; whereas Yukihara *et al.*[63] reported an over-response of 1.8%. These discrepancies need further attention, as has been the case for any other dosimeter. However, no change in the relative response has been reported by varying the energy and the angle of the incidence of the electron beams for energies 6, 9, 12, 16, and 20 MeV. The use of thin black plastic covers for protection of the OSL dosimeters from light did not pose any hindrance in their use as a surface dose detector for electron beam (with or without bolus) irradiation. Also, the integrated OSL response is not dependent on the temperature during irradiation in the range of 10°C to 40°C,[65] which is of great advantage for patient dosimetry.

**Table 3:** Energy dependence of Al2O3:C (0.3 mm thick and 7 mm diameter, wrapped in black tape of thickness 34 mg/cm^2^) to photon (at d_max_) and electron radiotherapy beams from Varian 21 EX linear accelerator[65]

*Radiotherapy beams*	*Relative response*	*Corrected relative response*
6 MV Photon	1.000	1.000
18 MV Photon	1.005	1.005
9 MeV Electron	1.019	1.000
20 MeV Electron	1.023	1.003

### OSL and RL of Al_2_O_3_:C for real-time/on-line mode for in vivo dosimetry of radiotherapy beams

Intrusion for the use of OSL for real-time/on-line dosimetry appears to stem from the studies of plastic scintillation dosimeters which have been found useful for real-time measurements of small radiation fields. The applications of small-volume water-equivalent plastic scintillation detectors include high-energy beam dosimetry,[66] ophthalmic plaque dosimetry,[67] dosimetry of stereotactic radiosurgery beams,[68] etc. The main problem in the use of plastic scintillation detectors has been the interference of Cerenkov radiation (called stem effect) produced in the optical fiber connecting plastic scintillation detector. For a compensation for the contribution from Cerenkov radiation, a second optical fiber of the same type and size without any scintillation detector is used. The presence of the second fiber complicates the system and limits its application at the sites with small gradients in dose distribution. The noise from the fiber becomes more problematic for an *in vivo* patient dosimetry when the positioning of the fiber changes during the measurements, especially for high-energy beams. The availability of OSL dosimeters of size similar to plastic scintillators has opened up a possibility of avoiding the use of a second fiber for real-time *in vivo* measurements.

**Figure 3:**
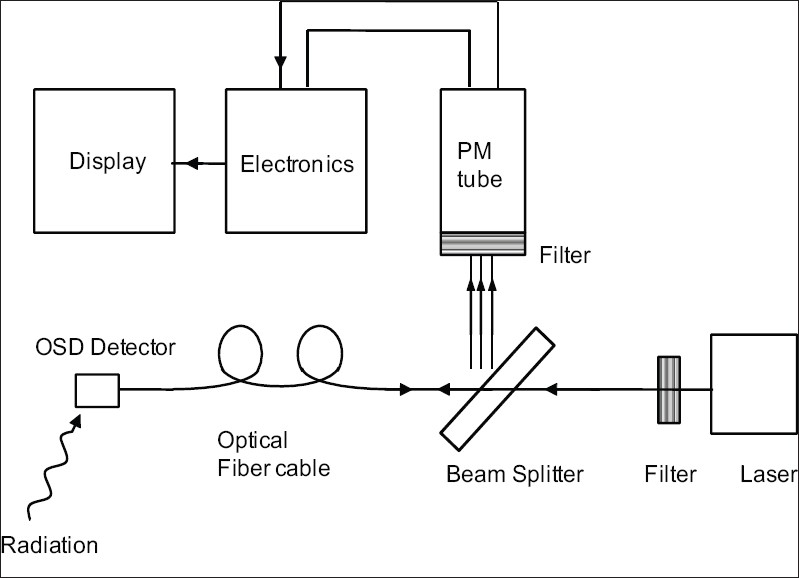
A simple schematic representation of real-time/on-line readout system and the recording of RL and OSL signals

Figure 3 shows the commonly used schematics of on-line measurements. A small-size (e.g., 1×1×2 mm^3^ or of length 2 mm and diameter 0.5 mm) OSL detector (Al_2_O_3_:C) with polished surfaces is coupled to an optical fiber cable. The other end of the fiber cable is coupled to a stimulating light source, usually a laser. The laser beam is transported to the Al_2_O_3_:C detector. The signal from the Al_2_O_3_:C detector is transported back along the same optical fiber and reflected by a dichroic color beam-splitter to reach the PM tube. The beam splitter selectively reflects the signal emitted by the Al_2_O_3_:C detector. It is thus possible to use a single fiber because the wavelength of the emitted light from the Al_2_O_3_:C detector is very different from that of the stimulating laser. The optical filters at the entrance of the PM tube and at the exit of the laser beam are so chosen that only the emitted light due to OSL reaches the PM tube and proper stimulation is provided to the detector probe. The output of the PM tube is suitably amplified and recorded through the associated electronicsand displayed. The OSL detector emits both RL and OSL of the same wavelengths. RL is emitted only during the irradiation and is a measure of the dose rate, whereas OSL signal measured at any time after an irradiation represents the total dose. This is the main principle, but there are many intricacies causing changes in the RL and OSL signals. These intricacies have been studied and suitable procedures for corrections have been established.

Aznar *et al.*[69] evaluated the properties and the performance of a real-time optical-fiber dosimetry system for radiotherapy photon beams of energies 6 and 18 MV by using a small (1×1×2 mm^3^) single crystal of Al_2_O_3_:C (cut from an OSL-grade cylindrical chip of 5 mm diameter and 1 mm thickness) connected through a 10 m long optical fiber made of PMMA core (Super Eska SK-40, Mitsubishi Rayon Co. Ltd., Japan). Studies on the reproducibility, energy dependence, dose rate dependence, field size dependence, depth and lateral dose distributions, and clinical test against TMS treatment planning system (Helax, Uppsala, Sweden) were carried out. Interference from Cerenkov radiation to RL in dose rate measurement was found to be up to 6%. The RL signal recorded with the beam-on time showed some initial (first few seconds of beam-on) variations. The RL reached a maximum value after a few seconds (within the first 3 seconds) from the start of an irradiation, followed by a near constancy when compared with the expected dose rate. However, it was concluded that the measurements of RL and OSL can satisfactorily verify the overall treatment delivery within an accuracy of about 5% that is needed in clinical dosimetry.[69] Since the wavelength of Cerenkov radiation (peaking around 365 nm) differs from the RL (peaking around 410 nm) emission, the beam splitter helped in the reduction of the contribution of Cerenkov radiation. This also helped to increase the signal-to-noise ratio but did not eliminate the stem effect completely. Edmund *et al.*[70] studied the effect of a pre-dose on the RL and OSL responses of Al_2_O_3_:C and found an inverse relation between OSL and RL, i.e., when RL increases, OSL decreases. To improve reproducibility and linearity, CW-OSL protocols were developed, in which a crystal-specific minimum pre-dose was found to be necessary for stabilization of the signal.

Andersen *et al.*[71] discussed two problems encountered in the RL/OSL method, viz., 1. interference from Cerenkov radiation (stem effect) and 2. nonlinearity of the RL response with dose rate due to changes in sensitivity and the presence of shallow traps. For pulsed linac beams of 6-MV photons from Varian Clinac 2300C/D, the stem effect problem has been solved by using a time-separation technique, as the RL signal from Al_2_O_3_:C has a lifetime of about 35 ms, whereas emission of Cerenkov radiation is prompt. For the sensitivity changes, an algorithm was developed which corrects the RL signal in a purely empirical way. With these corrections, the RL signals from Al_2_O_3_:C could be exploited to measure real-time dose rates with a time resolution of 0.1 second. Tests using an IMRT beam suggested that the method is fairly accurate and precise.[71] To overcome the interference of Cerenkov radiation, Gaza *et al.*[72] used POSL during an irradiation and the luminescence signal was alternated between periods of RL (between laser pulses) and a combination of RL and OSL together (during the laser pulses). The difference of the two signals gave only the OSL part free of RL, and this resulted in developing a measurement procedure and a correction algorithm for dose estimation at a speed of up to 2 seconds per data point in the range of the dose rates used in radiotherapy.

Recently, Edmund and Andersen[73] investigated the temperature dependence of the RL from probes of different sizes in the temperature range from 10°C to 50°C to further improve the accuracy of the system and extended it to both RL and OSL. Careful investigations revealed a change from −0.2% to 0.6% per °C with respect to the response at 25°C. It may be noted that Al_2_O_3_:C also has shallow traps related to −13°C to 37°C.[74] Change in temperature during both the irradiation and stimulation affected OSL, whereas RL was influenced only by the temperature during irradiation. If the temperature during an irradiation can be kept constant, the temperature effect on RL can be eliminated and the effect on OSL can be minimized by adopting the method of integration of OSL for longer times or longer delays. The mechanism related to the effect of temperature is attributed to the presence of shallow traps. Although it is possible to correct for these effects due to the changes in temperature, it has been recommended that calibration and actual measurements should be carried out at the same temperature. More recently, by using an Al_2_O_3_:C OSL probe of 0.5 mm thickness and length 2 mm attached to a 15 m long plastic (PMMA) optical probe of 1 mm diameter, a 6-MV photon beam, a solid water phantom, and a standard ionization chamber as a reference dosimeter, Damkjaer *et al.*[75] reported on two improvements, viz., 1. a stable calibration method which is independent of the reference radiation dose rate and 2. a correction for the effect of shallow traps. This appears to have ensured the satisfactory performance of OSL dosimeters using Al_2_O_3_:C for an *in vivo* on-line dosimetry.

### OSL of KBr:Eu for high-resolution real-time dosimetry of radiotherapy beams

Recently, Gaza *et al.*[76] explored a new possibility of overcoming the interference from Cerenkov radiation from the optical fiber cable for the use of OSL in real-time dosimetry by using KBr:Eu.[47] This material with its intense OSL emission peaking at 420 nm (characteristics of Eu ions) was chosen because of its very short luminescence lifetime as compared to the lifetime of 35 ms in Al_2_O_3_:C. For real-time dosimetry, a KBr:Eu crystal of the size of a few cubic millimeters was coupled to one end of a 5 m long optical fiber. The other end of the optical fiber was terminated at a standard FC connecter attached to an OSL reader via a coupler (arrangement similar to Figure 3). During the on-line measurements, as the irradiation and the measurements progressed, the OSL signal was the highest at the start of the laser stimulation which then attained equilibrium as the stimulation continued and did not decay to an instrumental background. Laser stimulation by a 658-nm diode laser was then applied periodically for a fixed interval (e.g., 20 ms), followed by a laser-off period (e.g., 20 ms). The equilibrium value attained during the optical stimulation was dependent on the dose rate and the laser power for the stimulation. When the stimulation was off, the luminescence decreased abruptly (without any afterglow) due to very short lifetime of the luminescent center. The difference (ΔOSL) between the peak value and the equilibrium value is related to the total dose accumulated during the interval between the two successive stimulations and hence provides a measure of the average dose rate. The duration, the frequency of stimulation, and the laser power for the stimulation were optimized. ΔOSL exhibited good linearity in the studied dose rate range, 10 to 500 mGy/s. KBr:Eu with Eu concentration of 1700 ppm was concluded to be a fast real-time OSL dosimeter with a small temperature dependence, but its application to routine dosimetry is yet to be explored.

## OSL in mammography and CT dosimetry

In recent times, concern has grown significantly on the need for a mammography screening in order to detect the early stages of breast cancers. However, concern has also grown for the risk from radiation, and this has necessitated the estimation of an average glandular dose which is usually derived from the entrance surface air-kerma, the penetrating power of x-rays (half-vale layer, HVL), and the compressed breast thickness. The breast composition is taken to be 50% fat and 50% glandular tissue. Usually, these measurements are done without any patient. In some countries (e.g., Sweden), there is a legal requirement to carry out direct patient measurements on a regular basis at least in some representative patients.[77] A tiny real-time OSL dosimeter appears to bea better dosimeter for in vivo measurements by overcoming the limitations of the ionization chambers and passive TLDs. Aznar *et al.*[61,77] evaluated the performance of a dosimetry system based on the RL and OSL responses of Al_2_O_3_:C similar to that developed for the *in vivo* dose measurements of radiotherapy beams. A system similar to that shown in Figure 3 was used with the only difference that two detectors (each of thickness 0.48 mm) and two measuring systems (instead of one used in conventional radiotherapy measurements) were used for the simultaneous entrance dose and exit dose measurements. One of the detector probes was used for the entrance dose measurement and the other for the exit dose measurement by placing them such that they do not overlap. The optical fiber cable also had a diameter of only 0.48 mm (excluding thin cladding) to match with the detector probe. The RL and OSL signals were recorded and corrected for the background signals recorded without any exposure. At such low x-ray energies, the interference from Cerenkov radiation is not encountered. Also, the sensitivity changes in the RL response are negligible at the low doses of interest in mammography. The photon energy dependence from 23 to 35 kV was within 18%, and the overall reproducibility for any exposure was within 3% in the studied dose range 4.5 to 30 mGy (with a linear response). During the *in vivo* measurements on patients, the presence of the probes did not significantly interfere with the image as the quality of diagnostic images was acceptable to the expert radiologists. The entrance and exit doses agreed (within 3%) with the values obtained by an ionization chamber. Thus the OSL/RL system appears very attractive for routine *in vivo* dose measurements in mammography.

In CT (x-ray–computed tomography), a CTDI (CT dose index) represents an average dose, and the measurements are only an approximation of the patient's dose. CTDI is usually measured with a pencil-shaped ionization chamber. In an interventional or perfusion CT, the dose could be best evaluated by a point dose measurement. In such situations, miniature real-time OSL detectors appear very attractive. It has been shown that for a spiral CT, the CTDI and point dose values are nearly the same for the measurement of surface doses; but for a perfusion CT, the dose is overestimated by a factor of 2 or more by the CTDI values in comparison with the point dose measurements.[78] Real-time measurement system based on OSL of KBr:Eu has also been evaluated, and its performance has been compared with a pencil ionization chamber.[79] It is evident that OSL dosimetry systems have a great potential for providing a point dose measurement as a valuable alternative to CTDI.

## Conclusion

Developments in the field of optically stimulated luminescence have increased at a very fast rate in the last few years. As evident from the increasing rate of publications, significant progress has been made not only in understanding the phenomenon but also in more effectively developing new techniques and materialsand upgrading the instrumentation and engineering aspects. Applications of OSL are no more limited to archeological and geological dating and to its use in computed radiography. Commercially available OSL systems have already become very popular for personal dosimetry. These have also found wide application in environmental monitoring, space dosimetry, and medical physics. OSL has specific advantages over the other available systems such as plastic scintillation dosimeters, diode detectors, MOSFET detectors, radiochromic films, TLDs, etc., in various clinical applications. At this point of time, Al_2_O_3_:C is dominating in all OSL applications, mainly because of its very high sensitivity; but in the very near future, some new materials with better characteristics are likely to become available. The present developments indicate that reliable real-time dosimeter systems based on OSL are likely to become commercially available in the near future for *in vivo* and point dose measurements.
